# Stabilization of a steerable sheath during retrograde access to antegrade-oriented branches in complex endovascular aortic aneurysm repair

**DOI:** 10.1016/j.jvscit.2020.03.014

**Published:** 2020-04-16

**Authors:** Charlotte C. Lemmens, Barend M.E. Mees, Michiel W. de Haan, Geert Willem H. Schurink

**Affiliations:** a Department of Vascular Surgery, Maastricht University Medical Center, Maastricht, The Netherlands; b Department of Radiology, Maastricht University Medical Center, Maastricht, The Netherlands

**Keywords:** Thoracoabdominal aortic aneurysm, Endovascular treatment, Branched stent graft, Steerable sheath

## Abstract

During branched endovascular aneurysm repair, cannulation of the visceral target vessels through antegrade branches and insertion of bridging stents are frequently done from an upper extremity access. A retrograde femoral approach is a challenging alternative when an antegrade approach is not preferred. Herein, we describe a technique to increase stability of a steerable sheath, using a single suture, for bridging antegrade-facing branches from a retrograde access. This technique secures the sheath's deflected tip and provides more pushability to the steerable sheath.

Femoral access during endovascular treatment of thoracoabdominal aortic aneurysms is often combined with an upper extremity (UE) access to cannulate and to insert bridging stents into the visceral target vessels. Particularly for stent grafts with antegrade branches, a UE access may be beneficial for antegrade catheterization.^1^ However, UE access is associated with occurrence of complications, such as stroke, pseudoaneurysm, hematoma, nerve compression, thrombosis, and distal embolization.^1^, ^2^, ^3^ A retrograde femoral access, using steerable sheaths or curved delivery sheaths, can be used as an alternative when a UE access is contraindicated.^2^^,^^3^ However, the insertion of stiff guidewires or bridging stents through a curved sheath tip may be challenging because of their rigidity. This report describes a new technique to increase the stability and pushability of a steerable sheath through a retrograde femoral approach during branched endovascular aneurysm repair (BEVAR). The patient has consented to publication of the details and images pertaining to the case.

## Case report

**Fig 1:**
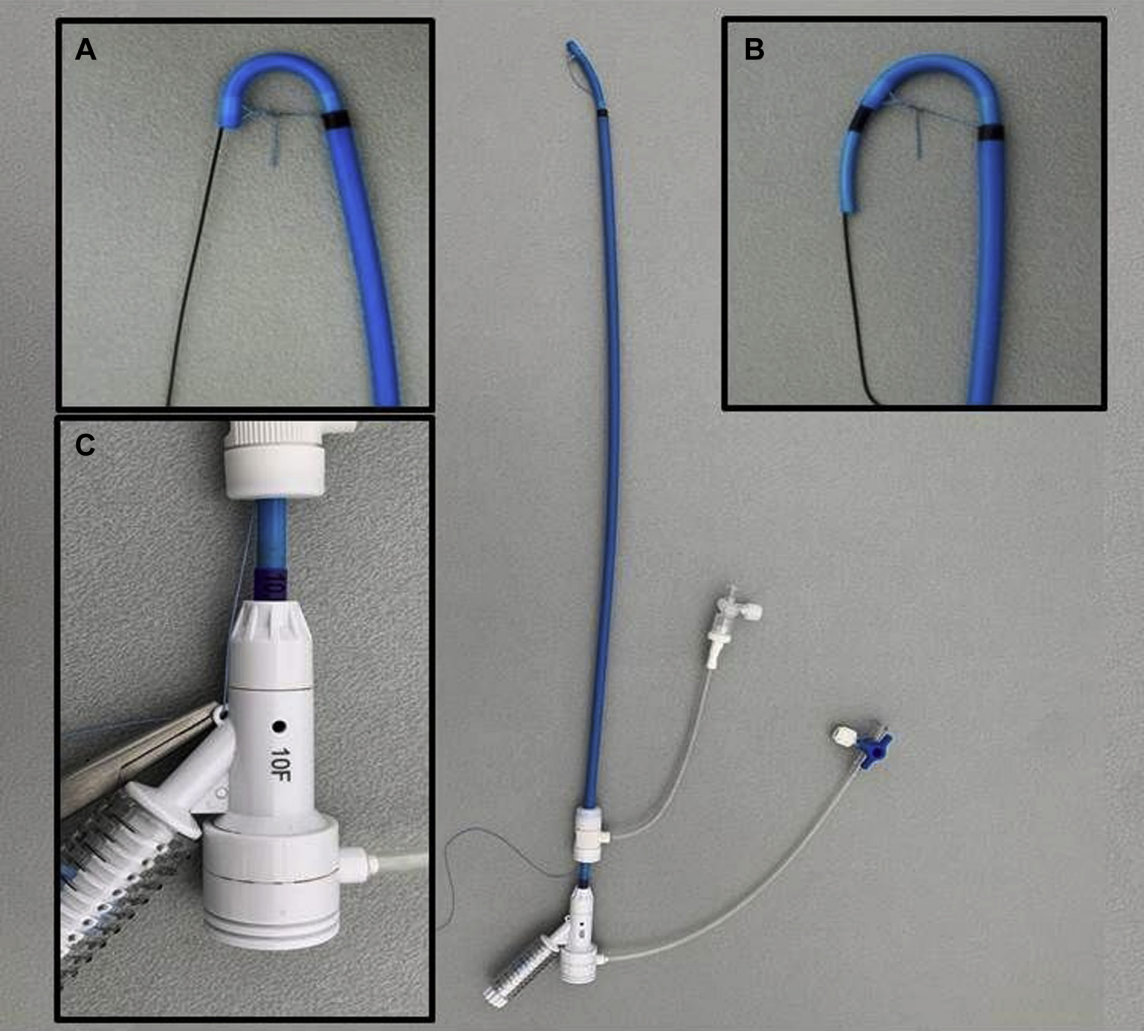
A 10F steerable Fustar sheath through a 14F Check-Flo sheath including the stabilizing suture. **A,** After the suture is pulled, the angle diameter is reduced. **B,** The sheath's tip is stabilized with the attached suture when an additional sheath is advanced through the curved steerable sheath. **C,** Handle of the steerable sheath with fixation of the pulled suture.

**Fig 2:**
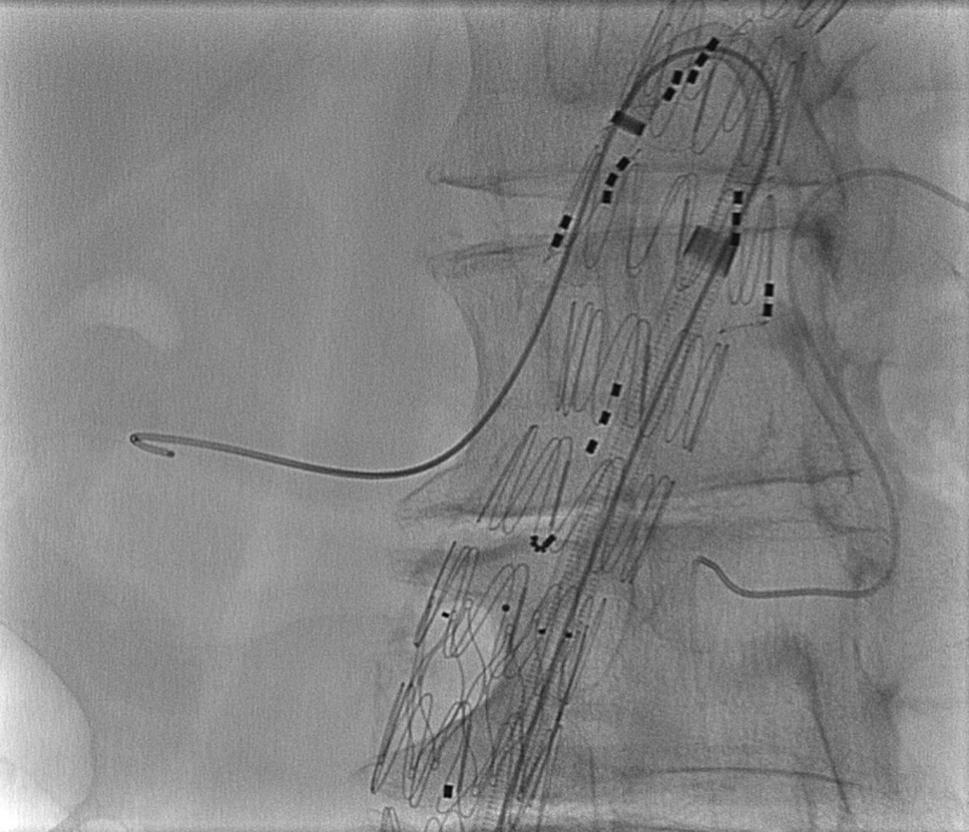
The steerable sheath and attached suture are inserted into a 14F insertion sheath. The sheath tip is curved to the preferred angle, and a wire is advanced through the branch into the target vessel. (Permission for use granted by Cook Medical, Bloomington, Ind.)

Our stabilization technique for retrograde catheterization of branches was first electively performed in a 64-year-old woman with a type II thoracoabdominal aortic aneurysm with a prior history of an open ascending and arch aortic repair with proximal reimplantation of the supra-aortic vessels and frozen elephant trunk. Bilateral percutaneous femoral access was obtained. On the right side, two ProGlide vascular closure devices (Abbott Vascular, Redwood City, Calif) were used. On the left side, a 5F sheath for a diagnostic catheter was inserted. Three thoracic stent grafts and a Cook Zenith t-Branch (Cook Medical, Bloomington, Ind) with four antegrade branches were deployed. Distal seal was established with a thoracic cuff just above the aortic bifurcation. The right femoral access was downgraded to accommodate insertion of a 14F 45-cm sheath (Check-Flo; Cook Medical) by pulling one of the ProGlide sutures. To catheterize the branches and corresponding target vessels, a 10F 55-cm Fustar steerable sheath (Lifetech Scientific Corp, Shenzhen, China) was used. A 2-0 90-cm braided polyester suture (Ti-Cron; Medtronic/Covidien, New Haven, Conn) was sutured at the inner curve of the sheath about 5 mm from the distal end, exactly at the location of the distal marker. The steerable sheath along with the attached suture was then introduced through the 14F sheath into the branched stent graft (Fig 1). After the steerable sheath was deflected toward the preferred angle, the end of the suture was pulled (Fig 1, *A* and *B*) and wrapped around the sheath's handle for a firm grip (Fig 1, *C*). The tip of the sheath was hooked into the respective branches for more stability. Subsequently, four branches and corresponding vessels were cannulated (Fig 2), and bridging stents (BeGraft Peripheral Plus; Bentley InnoMed GmbH, Hechingen, Germany) were placed over a Rosen wire (Cook Medical). If necessary, a 7F or 8F 70-cm Flexor sheath (Cook Medical) could be advanced through the branch into the target vessel. The downward traction of the suture secured the sheath's angle while sheaths or stents were advanced through the curved tip. The procedure was completed without complications. Completion angiography demonstrated no endoleak and patency of all stents. The patient recovered uneventfully, and postoperative computed tomography angiography confirmed patent target vessels and adequately positioned bridging stents.

## Discussion

**Fig 3:**
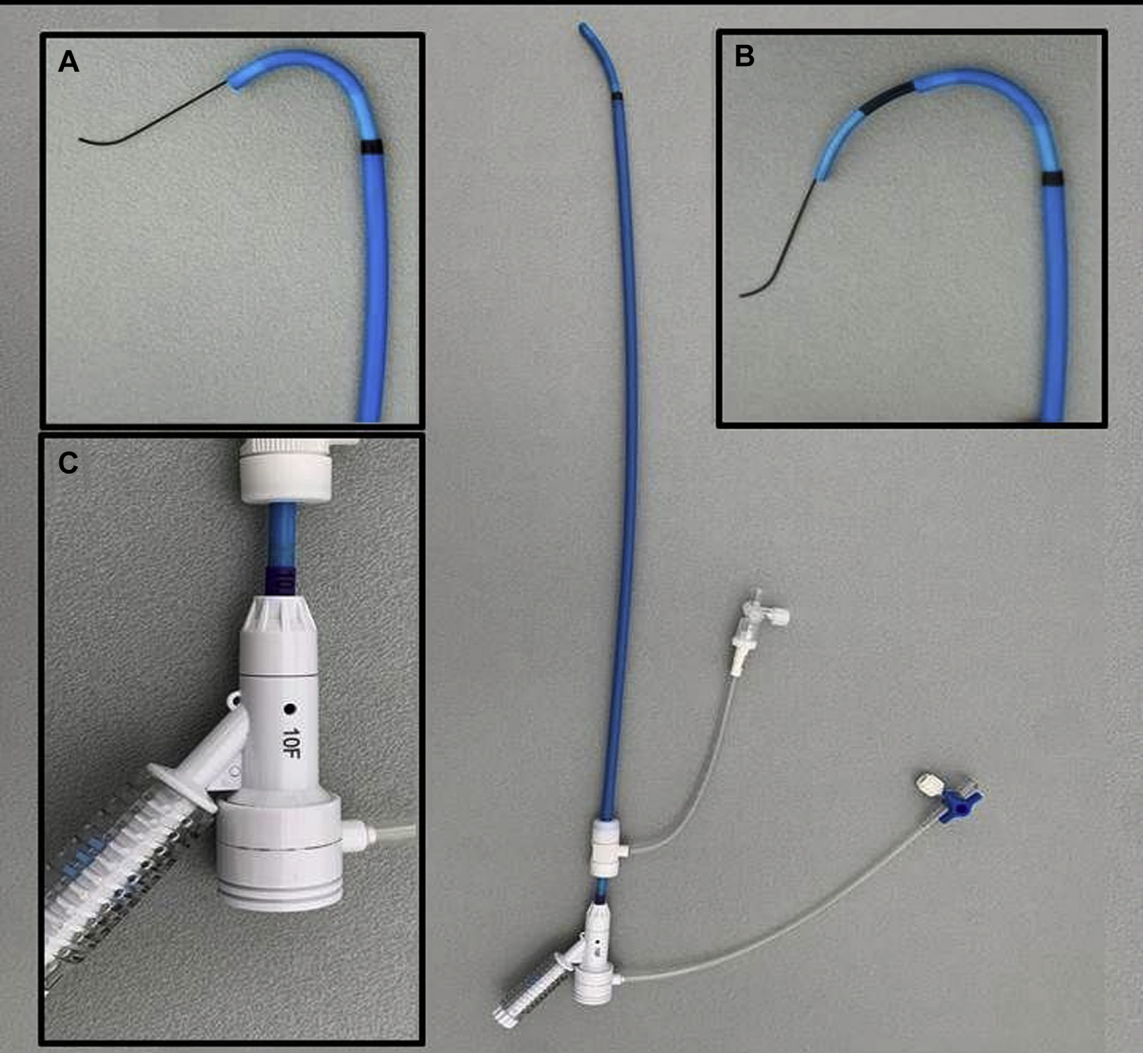
A 10F steerable Fustar sheath through a 14F Check-Flo sheath. **A,** The steerable sheath is shown with maximal curve. **B,** When an additional sheath is advanced through the steerable sheath, the curve diameter increases. **C,** Handle of the steerable sheath.

During BEVAR, a UE access has always been the preferred approach for catheterization of antegrade branches and their target vessels. However, there may be anatomic limitations to a UE access, such as a debranched aortic arch, frozen elephant trunk, severe tortuosity, shaggy arch, or thrombus in supra-aortic branches or in the aortic arch.^2^ Furthermore, a UE access is associated with stroke,^1^^,^^4^ particularly with a right or bilateral UE approach.^5^ To refrain from using a UE access, a retrograde femoral access has been described for target vessel cannulation and stenting during BEVAR. To adjust for the angulation from a retrograde approach, a steerable sheath can be used. This is a difficult technique because of lack of pushability and angle stability. Advanced iliac angulation can increase these difficulties. Therefore, we describe a new technique for stabilization of a retrograde steerable sheath for antegrade-oriented branches during BEVAR. A braided suture from the sheath's tip along the outside of the sheath can be pulled downward to secure the tip's angle. It prevents the tip from straightening out when stiff guidewires or rigid stents are advanced through the sheath (Figs 1 and 3). The range of angulation can even be increased as the external force of the supporting wire pulls the sheath tip farther downward. In theory, for our technique, any suture can be used. However, we propose the use of a braided suture as this does not stretch, as opposed to monofilament sutures like Prolene, resulting in a stable curve while a sheath or covered stent is being advanced into the branch.

Branched endografts usually have a relatively small diameter, so retrograde branch cannulation may benefit from a small curve for a better range of motion inside the endograft. A small curve can be provided by a steerable sheath,^2^ with the possibility of stabilizing and increasing its curve with a suture, as described in this paper.

Different techniques for retrograde cannulation of downward-facing target vessels or branches have been described previously.^2^^,^^3^^,^^6^^,^^7^ A steerable sheath for retrograde use can be created by looping a 0.014-inch guidewire or a suture through a flexible nonsteerable sheath.^6^^,^^7^

Despite the advantage to creation of a steerable sheath using cheap off-the-shelf materials, this technique is limited by the reduction of the inner diameter. The looped 0.014-inch guidewire or suture decreases the inner lumen of the 10F Fustar sheath, making the passage of a 7F or 8F sheath impossible.

With our technique, the full luminal diameter of the steerable sheath is preserved. Furthermore, because the suture is attached to the Fustar 5 mm from the tip, the free part of the tip can be hooked into the branch, providing for more stability. This is not possible with a looped guidewire or suture through the lumen of the sheath, as described by Mallios et al^6^ and Panuccio et al.^7^

Thus, the main advantages of our technique are increased stability and pushability, a small curve diameter, and an increased range of deflection.

A retrograde approach is associated with prolonged use of large access sheaths in the femoral arteries. As opposed to a UE access approach, the large femoral sheaths cannot be removed for early reperfusion. This may lead to lower extremity ischemia and subsequently even compartment syndrome, or it may increase paraplegia rate.^8^^,^^9^ Therefore, in our technique, we downgraded to a 14F access sheath to guide our steerable sheath, resulting in better distal perfusion.

Thus far, we have used this technique in 12 cases, in which an additional UE access has not been necessary because of failure of the retrograde approach. This technique has become our preferred approach for treating all BEVARs.

## Conclusions

This technique for retrograde cannulation of antegrade branches during complex endovascular aortic repair, using a steerable sheath and a braided suture, provides stability and pushability and facilitates retrograde insertion of bridging stents while securing the sheath's curved tip. This technique provides a stable alternative when a UE access is not preferred.
